# Assessing Children ‘At Risk’: Translation and Cross-Cultural Adaptation of the Motor Behavior Checklist (MBC) into Arabic and Pilot Use in the United Arab Emirates (UAE)

**DOI:** 10.3390/jintelligence10010011

**Published:** 2022-02-05

**Authors:** Maria Efstratopoulou, Hala Elhoweris, Abeer Arafa Eldib, Eleni Bonti

**Affiliations:** 1 Department of Special Education (CEDU), United Arab Emirates University (UAEU), Al-Ain 112612, United Arab Emirates; maria.efstratopoulou@uaeu.ac.ae (M.E.); halae@uaeu.ac.ae (H.E.); eldib@ese.gov.ae (A.A.E.); 2 First Psychiatric Clinic, “Papageorgiou” General Hospital, School of Medicine, Faculty of Health Sciences, Aristotle University of Thessaloniki, Ring Road Thessaloniki, N. Efkarpia, 54603 Thessaloniki, Greece; 3 Department of Education, School of Education, University of Nicosia, Nicosia 2417, Cyprus

**Keywords:** Motor Behavior Checklist (MBC), cross-cultural adaptation, UAE, physical education, behavioral problems

## Abstract

Children’s emotional, behavioral, and developmental problems can be properly identified and assessed based on observations from their teachers and parents. The Motor Behavior Checklist (MBC) was designed to assist classroom teachers and Physical Education (PE) teachers in assessing their students’ motor-related behaviors. The instrument has already been successfully translated and culturally adapted into six languages and used in a number of research studies internationally. The present study aimed to develop the Arabic version of the MBC checklist and proceed with the necessary cross-cultural adaptations for the use of the instrument in Arabic speaking countries and especially in United Arab Emirates (UAE) primary schools. The translation and cultural adaptation of the MBC was based on the ten-step process: forward translation of the original instrument; development of a synthesized version, back-translation; linguistic and semantic comparisons; back translators evaluation of divergent items; development of a synthesized version; based on the back translators’ suggestions; clarity assessment of the synthesized version by professionals (teachers); additional assessment of clarity indicators by a focus group of experts; and development of the final version. Results indicated a satisfactory level of agreement between the original and the back-translated versions, while nine items required minor adjustments and two items needed major adaptations and word replacements to clarify their content and be fully adapted into the UAE culture. In the pilot use, UAE teachers confirmed the clarity of the items in an 84% percentage. The final translated version’s overall content was found sufficiently compatible with the original version of the instrument. The study highlights the importance of a rigorous translation process and the process of cultural adaptation.

## 1. Introduction

### 1.1. The Manifestation of NDD in School-Aged Children

Neurodevelopmental disorders (NDD) constitute a group of disorders that commonly emerge during childhood or adolescence and usually affect behaviors that are significant for normal interactions, ranging from school to social occasions (American Psychiatric Association 2013). NDD include Intellectual Disability Communication Disorders, Autism Spectrum Disorder (ASD), Attention-Deficit/Hyperactivity Disorder (ADHD), Specific Learning Disorder (SLD), and Motor Disorders. They manifest early in development, often before school entry age, affecting the child’s personal, academic, and social functioning. The range of NDD developmental deficits may vary from very specific limitations of learning or control of executive functions to global impairments of intelligence or social skills (American Psychiatric Association 2013). Research indicates that Neurodevelopmental Disorders in children may co-occur and there are some common behavioral, cognitive, andemotional characteristics, which often lead to a challenging or invalid diagnosis or a misdiagnosis (Jensen et al. 2001). It is often the case that many children with NDD enter elementary school without having received a formal diagnosis. As a result, their cognitive/learning, language, attentive, emotional, and/or behavioral difficulties (EBP) often lead them to ‘school failure’ along with low self-esteem issues, while in certain cases, they may face more severe psychological or psychiatric conditions as adults (Eisenberg et al. 2000).In addition, unlike learning or cognitive assessment, which can be attained through direct assessment, children’s emotional and behavioral competencies can only be properly identified and described mainly based on the reports of others (Efstratopoulou et al. 2015).

### 1.2. Students of Determination in United Arabs Emirates

The number of pupils with a disability enrolled in the emirate’s schools increased by more than 3500 in the past year, the Knowledge and Human Development Authority revealed. The results of the 2018–2019 inspections by Dubai’s private schools’ regulator revealed that pupils with physical and intellectual disability account for six per cent of the 278,794-strong school population. Inspections also revealed that 71 per cent of schools provide a good or better quality of education for disabled pupils, also referred to as ‘people of determination’ by the UAE government.

Early assessment is one of the most important components in the UAE education system according to the new National Standards for Education presented by the Ministry of Education. The new strategy of the Ministry of Education (MOE) focuses on supporting early assessment and professional development and training for teachers. In this direction, the translation and cultural adaptations of valid assessments tools (like the MBC for children) that can be used by professionals (teachers/physical educators) in schools settings is of high importance.

### 1.3. Teachers and Parents’ Involvement in the Assessment Process in Children

Researchers have recognized the importance of parents and teachers’ roles in obtaining a more holistic and valid assessment of children’s emotional and behavioral functioning. Especially, as regards the evaluation of EBP encountered by children with ADHD (e.g., inattention, lack of concentration, impulsivity, hyperactivity, learning problems, etc.), teachers and parents are often considered as the principal agents of the assessment process (Gardon 2012). More specifically, with the use of several assessment scales, they are asked to rate their students/children’s behaviors across a variety of settings (e.g., home, school, athletic activities, play, etc.) in common, everyday circumstances (Paiano et al. 2019). These behaviors are often related to problems in academic achievement, social maturity issues, and family relationships, which are usually evident in children with NDD (Haack et al. 2019).Furthermore, teachers, in particular, are considered as the most representative and valid source of information regarding their students’ behavioral problems, since they interact with the children on a daily basis and within the school environment, where specific behavioral patterns are expected (Gardon 2012).

### 1.4. PE Teachers’ Role in the Identification of Emotional and Behaviour Problems in Children (EBP)

A significant body of research has indicated that Physical Education (PE) teachers, in particular, are among the most appropriate educators in the assessment process, since they have the knowledge and skills to better identify the warning signs of abnormal, especially motor-related behaviors. Therefore, they can provide valuable information about the overall development and EBP manifestation of their students (Kashani et al. 1997; Mol Lous et al. 2002). More precisely, as Kashani et al. (1997) have argued, evidence for the presence of externalizing and/or internalizing behavioral symptoms can be better obtained in multiple active situations during PE classes and team games. In addition, specific problematic behaviors (e.g., attention, contact, learning, or mood difficulties) can be systematically observed during standardized play procedures (Mol Lous et al. 2002). Hence, PE lessons and group play situations offer multiple opportunities for observing a child while s/he engages with his/her classmates in various settings (e.g., inside or outside the classroom, at the gym, during group activities or games) (Efstratopoulou et al. 2015).

### 1.5. School-Based Behavioural Evaluation Instruments

The majority of school-based behavioral assessment tools are structured for use by school psychologists and/or counsellors, often use mental health terminologies, are time-consuming, and are not intended for use by teachers or physical educators in school settings. Data obtained through such rating scales are interpreted mainly by medical professionals and might lead to an official diagnosis. Thus, psychotherapeutic treatments and/or psychiatric therapy usually follow such diagnoses. However, teachers need behavioral assessment tools that are easy to use and interpret, to monitor the effectiveness of their supporting strategies and educational approaches in-class or school-based behavior support plans. School-based behavior rating scales must be valid, reliable, and concise screening tools, which lead to the early identification of children ‘at risk’ of developing behavioral, NDD, or other disorders. In addition, they should be available for use by classroom teachers and school staff and should be integrated into the mainstream educational system regularly (i.e., they could be administered to all students during their first years of primary education). Furthermore, such tools should also serve as a means for additional, more detailed assessments of students who meet the ‘at risk’ criteria, to safeguard that they will eventually receive the appropriate intervention at an early stage, thus preventing secondary problems, which usually accompany NDD, as the child grows older. Finally, they might also serve as a measure of the effectiveness of the intervention programs designed for these children, through the pre-test (prior intervention) and post-test (following intervention) result analysis process (Efstratopoulou et al. 2015).

### 1.6. The Motor Behaviour Checklist (MBC)

The Motor Behavior Checklist (MBC; Efstratopoulou et al. 2012b) is a practical, easy to administer, useful, and valid measure for observing the motor behavior of children aged between 6 and12 years, and for screening and assessing children with EBP problems and possible underlying disorders (e.g., motor-development problems, Autism Spectrum disorders, ADHD, learning difficulties, etc.) in the school environment. Its first version was standardized in the British primary school-age population (initially including 150 items). In its final version, it includes 59 items describing observable ‘problematic’ behaviors. The higher the total score recorded the more indicative of possible underlying problems. The instrument can provide separate scores for each of the seven factors and total externalizing/internalizing behavior scores. Externalizing behaviors include rule-breaking (seven items), hyperactivity and impulsivity (14 items), and lack of attention (10 items). Internalizing behaviors include low energy (four items), stereotyped behaviors (two items), lack of social interaction (10 items), and lack of self-regulation (12 items).

The MBC can be completed by the class teacher or the physical educator (provided that he/she knows the child well enough (i.e., more than six months)), using a 5-point Likert scale ranging from never (0) to almost always (4) indicating the frequency of the observed behavior (Efstratopoulou et al. 2019). The MBC does not require the physical presence of the child during administration, and data can be collected even via online procedures. The checklist cannot be solely used as a diagnostic tool but can be part of a broader assessment battery and is a quick and cost-effective screening tool/measure for the early identification of emotional–behavioral symptoms/difficulties in typical school-aged children, who are often left unnoticed because they are underdiagnosed or misdiagnosed. Finally, administration and completion of the MBC checklist do not require verbal skills on the child’s part and can provide a detailed individual profile on different areas of the child’s development (e.g., social skills, self-regulation, aggressiveness, hyperactivity, etc.), while assessing deviant behaviors in school settings (Efstratopoulou et al. 2015).

### 1.7. Psychometric Properties of MBC

Research on the psychometric properties of the instrument indicates that the MBC for children is a content-homogeneous instrument that has a strong correlation and is highly stable (Efstratopoulou et al. 2012a). More specifically, the internal consistency coefficients ranged from 0.82 to 0.95, reproducibility was 0.85 to 0.90 according to intraclass correlation coefficients (ICC), and concordance was also 0.75 to 0.91. Discriminant and convergent validity of the MBC scale were established, and it has been proven that MBC can provide valid ratings on externalizing and internalizing behaviors of school-aged children (Efstratopoulou et al. 2012b).

Although the MBC for children is not a diagnostic tool itself, it can provide valid complementary information on attentional, emotional, and developmental problems in children when used by physical educators in school settings. It is a new practical and useful measure for assessing externalizing and/or internalizing problems in elementary school-age children and could be used for various educational purposes, including research projects and intervention programs. Hence, the MBC can be considered as an effective method for assisting teachers and physical education instructors in referring students for a more comprehensive assessment, as well as for collecting data from children and youth as part of the clinical investigation process. This is because the behavioral patterns of children with ADHD and ASD can be more clearly expressed in social contact environments with different stimuli than in the classroom environment where stimuli are closely regulated, and students are expected to obey more strict rules of behaviors (Efstratopoulou et al. 2012b).

### 1.8. Previous Cross-Cultural Adaptation of MBC in Other Countries

Even though in the relevant literature there are contradictive views regarding the procedures that are necessary for a cross-cultural adaptation of assessment scales, scholars agree that the process must go beyond simple translation, since translation alone does not ensure the instrument’s reliability and construct validity (International Test Commission 2017). For example, Borsa et al. (2012) have suggested a six-step process of cross-cultural adaptation. These steps include (1) translation of the instrument from the source language into the target language; (2) synthesis of translated versions; (3) synthesis evaluation by expert judges; (4) evaluation of the instrument by the target groups; (5) back-translation; and (6) a pilot study. In addition, the authors also emphasized the importance of assessing the factorial structure of the instrument to confirm its stability concerning the original document. Alternatively, Mondrzak et al. (2016) used the guidelines proposed by the ‘Task Force for Translation and Cultural Adaptation of the International Society for Pharmacoeconomics and Outcomes Research’. This 10-step process can be described as follows: preparation, forward translation, reconciliation of different translations into a single version, back-translation, back-translation review, harmonization, cognitive debriefing, review of cognitive debriefing results and finalization, proofreading, and final report. Wild et al. (2005) have also proposed a similar process.

Previous studies on the evaluation of the psychometric properties of MBC for children have revealed that the MBC is a content-homogeneous instrument, with high temporal stability and high interrater agreement that can provide useful and reliable ratings on behavioral and emotional problems in children, especially when used by PE teachers in school settings (Efstratopoulou et al. 2012a, 2012b, 2013, 2015) (Table A1).

Up to date, the Motor Behavior Checklist (MBC; Efstratopoulou et al. 2012a) has already been translated into six languages (Greek, Polish, Urdu, Czech, Chinese, Brazilian/Portuguese) and has been used in several studies (Paiano et al. 2019; Wood and Efstratopoulou 2020; Efstratopoulou et al. 2017).

## 2. The Present Study

Recognizing the need for incorporating such an innovative screening tool (MBC) in the area of early diagnosis of neurodevelopmental, behavioral, and developmental disorders in the Arabic population, the researchers in the present study followed the ten-step procedure suggested by Wild et al. (2005) and Mondrzak et al. (2016), to translate and culturally adapt the MBC to the Arabic language and UAE culture. The main aim of the study is to provide a culturally validated version of MBC in the target language, which will assist clinicians and multidisciplinary groups working in the field of Special Education in carrying out more accurate, valid, and complete screening procedures and diagnoses of students, mainly those who might be ‘at risk’ of NDD, in the United Arab Emirates.

## 3. Method

Research that crosses linguistic and cultural boundaries necessarily requires direct attention to the use of language and cultural factors where verbal expressions, comprehension, or both are involved at any level in the systematic collection of data expected to exhibit comparable reliability and validity across the linguistic and cultural boundaries.

Idiomatic criteria were used to assess the equivalence with the new version. The analysis used and the estimation of contextual and cultural sensitivity was based on culture-loaded phraseological expressions that were used to exhibit strong contextuality. The structural equivalence of the list (and each sub-factor) was demonstrated between native speakers of the target language. It was important to test the measurement equivalence between native speakers of the source language and the target language. If the latter is not supported, the underlying reasons and cultural factors causing the lack of equivalence were further discussed.

### Characteristics of the Sample

The Physical Education (PE) teachers who participated in this study were native (Arabic) speakers with a specialization in Adapted Physical Education (APA) for children with disabilities. The criteria used for the selection of the professionals who participated in the Focus Group were: (a) To be native Arabic speakers, (b) To have at least four years of teaching experience in public primary schools in UAE, (c) To have a specialization in APA and knowledge of the characteristics of children with disabilities and of behavioral management interventions. For our sample, the professionals were all native speakers, had MN = 5.6 (SD = 1.2) years of teaching experience in Primary Schools in UAE and they all had a Degree in Physical Education with a concentration in Adapted Physical Activity (from the United Arab Emirates University). In addition, all of them had practical experience working in public (both mainstream and special) schools in the UAE, with children with disabilities.

**Figure 1:**
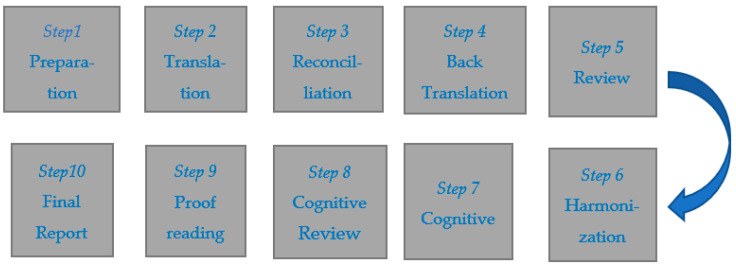
Steps involved in the translation and cross-cultural adaptation process of the MBC.

The translation and cross-cultural adaptation processes used in this study, as previously mentioned, followed the major guidelines suggested in the works of Wild et al. (2005) and Mondrzak et al. (2016). The process included ten steps, as illustrated in Figure 1 and Table 1. The Ethics committee of United Arab Emirates University (UAEU, Protocol No ERS_2021_7335) approved the project.

## 4. Results

The comparison between the back-translated version and the original version (step 4) revealed that 44 items were translated at a satisfactory level (78%). Fifteen of the items (22%), which were considered divergent, were forwarded to the back-translator, along with the original version, for further consideration. The back-translator characterized nine of these items as ‘semantically identical’––and, therefore, acceptable––, but commended on the other six items, setting up step 5 (Table 2). Based on the back translators’ comments, a further synthesized version of the instrument was developed (step 6). The next step (cognitive debriefing) revealed that 84.7% of the items were comprehensive and cognitively equivalent (i.e., 71% had a mean score of 2.87, while 15 items 18.6% had a mean score of 2.35). Nine items were considered partially clear (i.e., three items with a mean score of 2.5, three items with a mean score of 2.05, and two items with a mean score of 2.01). The overall results of the back-translated version analysis, along with the modifications proposed by the focus group (step 8) are illustrated in Table 2.

More specifically, most of the items were considered clear by the focus group and, therefore, required no alterations. Items 28 and 42––included in the hyperactivity factor––were characterized as ‘not clear enough’ and, therefore, the focus group and the back translator expert proposed major adaptations. These items were modified based on the recommendations from the focus group, to make sure that professionals in school settings in the UAE can observe and record these specific behaviors in their students during class activities and/or during free play situations. The rest of the items either remained the same as in the preliminary translated version or needed minor modifications, which, in most cases, was either the addition/replacement of a verb or the addition of an example to clarify the meaning and make sure that the rater is satisfactorily assessing students’ externalizing and internalizing behaviors in school settings.

## 5. Discussion

The present study described the processes followed during the translation and cultural adaptation of the MBC into the Arabic language. The new Arabic version of the MBC was developed to provide class teachers and physical education teachers in the UAE, with a practical, easy to administer, useful, and valid assessment tool to observe and record the motor behavior of their 6 to 12-years-old students. As aforementioned, according to Gardon (2012), teachers are considered as the most representative and valid source of information regarding their students’ manifestation of behavioral problems. Supported by valid and reliable assessment instruments, professionals in education can provide detailed reports on children’s deviant behavior observed in natural conditions of interaction and competition, which are rarely considered in evaluation protocols.

Educational professionals can use the MBC for screening and assessment purposes of children with emotional and/or behavioral disorders (EBP) and/or developmental disorders, during class activities physical activity classes and free-play situations. As research suggests, EBP are often highly prevalent among children and adolescents with neurodevelopmental disorders (e.g., autism spectrum disorder (ASD), Attention-Deficit/Hyperactivity Disorder (ADHD), Specific Learning Disorder (SLD), etc.) (Eisenberg et al. 2000). More specifically, it has been found that the specific behavioral patterns of children with ADHD and ASD can be more clearly expressed in social contact environments, such as free-play or sports, where stimuli are more closely regulated and students are expected to obey more strict rules of behaviors, rather than in the typical classroom environment (Efstratopoulou et al. 2012b). In addition, as Efstratopoulou et al. (2019) have pointed out, a detailed assessment, performed by both professionals and teachers using multiple instruments, can safeguard a proper identification of behavioral changes and/or developmental delays.

Further discussing the importance of cultural adaptations made in this study concerning the Arabic version of MBC for use by PE teachers and professionals in school settings, we need to point out that all adaptations made, were based on recommendations from professionals in UAE who are working with students in primary school settings. The professionals, who participated at the focus group, indicated that there was a specific need for some of the items to reflect the cultural environment in schools, taking into consideration that not all students’ deviant behaviors are easy to be observed in a structured class environment in UAE schools. Furthermore, the significance of cross-cultural adaptation of the MBC for use in different countries has also been demonstrated in previous studies (e.g., Paiano et al. 2019; Efstratopoulou et al. 2012a). Overall results are in accordance with other studies, which have also revealed that, based on the synthesized version, the translation and back-translation processes are adequate methods for translating and culturally adapting instruments, without major distortions (Mondrzak et al. 2016; Wild et al. 2005; Borsa et al. 2012; Gjersing et al. 2010; Mattos et al. 2006).

### Practical Implications and Recommendations for Future Research

Considering the above, the newly translated and culturally adapted version of the MBC, as described in this article, will comprise a useful tool for the assessment of children at risk of EBP and/or NDD, by physical education teachers and/or classroom teachers in the UAE. Furthermore, results revealed a satisfactory level of agreement between the original and back-translated versions, with 78% of exact equivalence between the translated items and 12% of terms requiring minor adjustments. In addition, clarity assessment using reports from teachers revealed an 84% agreement with the draft version of the MBC. The synthesized version of the instrument required modifications to ensure semantic and cultural adequacy in relation to the original version. Minor adaptations based on recommendations from professionals in UAE participating at the focus group, indicated that there was a need for some items to reflect the cultural environment in schools taking into consideration that not all students’ deviant behaviors are easy to be observed in a structured class environment in UAE schools. For example, for the items 19 and 25 (behaviors that are connected with lack of social skills), the focus group suggested the addition of the phrase: *when asked to do so* (which is missing from the original English version of the MBC checklist) but was proposed by professionals/educators in UAE) with the explanation that these social behaviors are not observable in UAE schools settings unless there is a specific request from the educators (class teachers/PE teachers).

It was also mentioned that due to schools’ rules and maybe cultural components, children in schools do not have the flexibility to express in different ways in a structure class environment unless they were asked or motivated to do so. In general, the Arabic version of the instrument showed adequate indicators of semantic equivalence following the steps of initial translation, back-translation, and clarity assessment by professionals and by the focus group.

Future research is needed to assess the psychometric properties (mainly the validity and reliability) of the new Arabic version of the MBC using a large sample of primary school-aged children from UAE schools rated by their teachers and physical educators in school settings. More specifically, future studies need to collect data from typical and clinical samples of children from UAE primary schools to assess the psychometric properties of the checklist and to ensure that the Arabic version of MBC is a new valid and reliable assessment instrument to support teachers, physical educators, and special educators in their role in UAE schools. In addition, evidence of validity should be demonstrated through multiple informants. A study currently in progress, by our group, has started to assess the psychometric properties of the translated version of the checklist.

Hence, the new instrument fills a gap in the evaluation process of students in sports and free-play situations. Moreover, it can help schoolteachers and physical educators to better understand and effectively deal with their students’ behavioral profiles, especially those with behavior problems compatible with NDD. Consequently, we highlight the importance of the cross-cultural adaptation of instruments for use in different countries, as demonstrated by previous works and performed in our study.

## 6. Conclusions

The cross-cultural adaptation and translation processes used in this article allowed the formulation of an Arabic version of the Motor Behavior Checklist for children (MBC; Efstratopoulou et al. 2012b) that will enable physical education teachers to evaluate their students’ behavioral aspects in sports and free-play situations. The Arabic version of the MBC was produced following rigorous translation and cross-cultural adaptation procedures. Results from the pilot use of the MBC in the UAE indicated that there is a satisfactory level of agreement between the original and the back-translated versions. The final translated version’s overall content was found sufficiently compatible with the original version of the instrument and was proven to ensure a more complete and comprehensive evaluation process. Teachers, PE teachers, and educational professionals will be able to provide valid reports on students’ behaviors, observed in natural school settings of interaction and competition, such as sports and free-play situations, which are rarely considered in evaluation protocols. Moreover, it can help schoolteachers to better understand and effectively deal with their students’ behavioral profiles, especially those with behavioral problems compatible with NDD. Future research is required to further assess the psychometric properties of the new Arabic version of the MBC using a larger sample of primary school-aged children from UAE schools rated by their teachers and physical educators in school settings. Therefore, we highlight the importance of the cross-cultural adaptation of instruments for use in different countries, as demonstrated by previous works and performed in our study.

## Figures and Tables

**Table 1:** Detailed description of the translation and cross-cultural adaptation process of the MBC.

Steps	Actions
**1**	**Preparation:** The project manager (who is also the MBC developer), recruited three key persons from the target country to work on the project. Permission for translation was acquired. The instrument developer provided information and clarifications on the conceptual basis of the instrument items for use by the translators. A key person from the target country worked closely with the project manager for the preparation of the translation process.
**2**	**Forward Translation:** Three professionals specializing in Special Education—native speakers of the target language (Arabic), with proficiency in English, independently translated the instrument into Arabic. The project manager worked closely with the translators to provide background information about the conceptual basis of the instrument and/or the particular wordings to be used in the items.
**3**	**Reconciliation of the forward translations into a single forward translation:** After completion of the individual translations, the translators compared the different versions of each item to reach a consensus, aiming at the most appropriate cultural adjustment and resolving possible discrepancies.
**4**	**Back translation of the reconciled translation into the source language:** The instrument was back-translated into the source language by an English teacher with proficiency in both the English and the Arabic language (i.e., an English native speaker living in the UAE).
**5**	**Back translation review:** To ensure the conceptual equivalence of the translation, the project manager and the key in-country person reviewed the back translation to identify any discrepancies or problematic items and to decide upon the best linguistic and semantic match between the wordings in the two versions. A professor of Arabic Language worked on the Arabic version during the back translation review procedure.
**6**	**Harmonization between all translated versions and with the source version:** This is an additional quality-control step to further ensure that all linguistic or conceptual discrepancies are resolved. Thus, based on the comments of the back-translator, the authors designed a new synthesized version (aggregation of the global data set).
**7**	**Cognitive debriefing:** to assess the level of comprehensibility and cognitive equivalence of the final translated version, four native speakers of the target language, specializing in the target area (i.e., Physical Education (PE) teachers *, working with school-aged children), evaluated the translated instrument. Additional issues causing confusion were resolved during this phase.
**8**	**Cognitive debriefing review:** The project manager reviewed the results from the cognitive debriefing and identified translation modifications necessary for improvement. Partially clear items were analyzed by a focus group composed of three Physical Education (PE) teachers *. Following agreement on changes between the project manager and the key in-country persons, the translation process was finalized.
**9**	**Proofreading:** The finalized translation was proofread to check and correct any remaining spelling, grammatical, or other errors. The final suggestions and corrections were sent for approval by the author of the original version.
**10**	**Final Report:** The project’s Principal Investigator developed the final report, which included a full description of the methodology used, along with an item-by-item representation of all translation decisions undertaken throughout the process. Finally, the author’s comments were analyzed, and the final version of the instrument was produced.

* The PE teachers participated in this study were native (Arabic) speakers with specialization in Adapted Physical Education (APA) for children with disabilities.

**Table 2:** Comparison between the original version of the MBC checklist and the translated and back-translated versions with semantic adaptation.

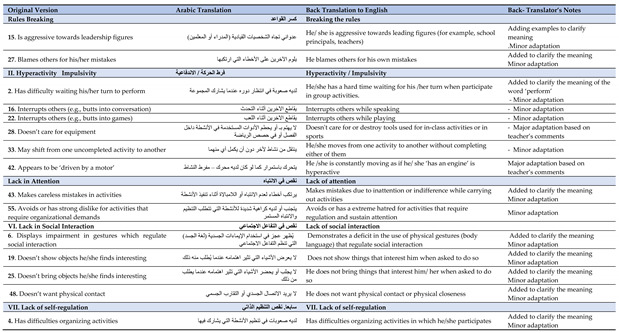

## Data Availability

The data presented in this study are available on request from the corresponding author due to privacy issues.

## Appendix A

**Table A1:** The Arabic Version of MBC after translation and cultural Adaptations.
